# Dry Matter Intake and In Vivo Digestibility of Grass-Only and Grass-White Clover in Individually Housed Sheep in Spring, Summer and Autumn

**DOI:** 10.3390/ani11020306

**Published:** 2021-01-26

**Authors:** MaryAnne Hurley, Eva Lewis, Marion Beecher, Brian Garry, Christina Fleming, Tommy Boland, Deirdre Hennessy

**Affiliations:** 1 Teagasc, Animal & Grassland Research and Innovation Centre, Moorepark, Fermoy, P61 P302 Co. Cork, Ireland; Maryanne.hurley@teagasc.ie (M.H.); Marion.beecher@teagasc.ie (M.B.); brian.garry@teagasc.ie (B.G.); Christina.fleming@teagasc.ie (C.F.); 2 School of Agriculture and Food Science, University College Dublin, Belfield, D04 V1W8 Dublin 4, Ireland; tommy.boland@ucd.ie; 3 Devenish Nutrition Limited, Lagan House, 19 Clarendon Road, Belfast BT1 3BG, UK; eva.lewis@devenish.com

**Keywords:** digestibility, *Lolium perenne*, season, sheep, *Trifolium repens*

## Abstract

**Simple Summary:**

Feed intake and the digestibility of that feed are key drivers of animal production from grazed forage. This study compared the digestibility and voluntary dry matter (DM) intake of grass-only and grass-white clover (grass-clover) forage in individually housed sheep. The study was a Latin square design, repeated in spring, summer and autumn in 2017. Grass-clover and grass-only forage was harvested daily and offered *ad libitum* to 6 individually housed wether sheep per treatment. Digestibility of the forage DM, organic matter (OM), neutral detergent fibre (NDF) and acid detergent fibre (ADF) was determined using the total faecal collection method. Dry matter intake was similar on both forage types. White clover inclusion increased forage crude protein concentration in autumn and reduced NDF concentration in the offered forage, resulting in increased nitrogen intake in autumn and reduced NDF intake in all seasons. Grass-clover swards had a significantly greater OM and DM digestibility compared to grass-only swards. This could potentially result in increased animal production from grass-clover swards compared to grass-only swards.

**Abstract:**

Intake and digestibility are key drivers of animal production from grazed forage. The objective of this study was to compare the in vivo digestibility and voluntary dry matter (DM) intake of grass-only and grass-white clover (grass-clover) forage in individually housed sheep. This study was a Latin square design, repeated on three occasions in 2017: Spring (27 March–29 April), summer (19 June–22 July) and autumn (4 September–29 September). Grass-clover and grass-only swards were harvested daily and offered *ad libitum* to 6 individually housed wether sheep per treatment per period. Digestibility of DM, organic matter (OM), neutral detergent fibre (NDF) and acid detergent fibre (ADF) were determined using the total faecal collection method. Dry matter intake was not significantly different between treatments. White clover inclusion increased forage crude protein concentration in autumn (*p* < 0.001) and reduced NDF concentration in the offered forage (*p* < 0.001), increasing nitrogen intake per sheep in autumn (*p* < 0.001) and decreasing NDF intake per sheep in autumn (*p* < 0.001). Grass-clover swards had a significantly greater OM and DM digestibility compared to grass-only swards (*p* < 0.05). This could potentially result in increased animal production from grass-clover swards compared to grass-only swards.

## 1. Introduction

Pasture is the primary and lowest-cost source of ruminant feed in temperate pasture-based systems [1,2,3]. In Ireland, approximately 84% and 82% of the diet, on a dry matter (DM) basis, of Irish sheep and dairy cows in intensive grazing systems, respectively, comes from pasture [4,5]. The digestibility of pasture reflects the energy available for intake, which is a measure of the overall pasture quality [6,7], affecting the voluntary DM intake (DMI) and animal performance [8,9] of grazing livestock.

Perennial ryegrass (*Lolium perenne* L.) is the most widely sown agricultural grass species in Ireland and the UK [10,11], typically selected for its high growth potential and high nutritive value [12]. The incorporation of white clover (*Trifolium repens* L.) into intensive pasture-based production systems provides many advantages in terms of feed value and animal performance, and can potentially contribute to improving the sustainability of pasture-based ruminant production systems [13,14]. Incorporating white clover in perennial ryegrass swards can increase the metabolisable energy content of the sward [15] due to its lower fibre concentration reflecting the absence of structural components such as stem and sheaths [16]. A recent meta-analysis by Johansen et al. [9] reported grass species with similar organic matter digestibility (OMD) resulted in comparable DMI and milk production, but when legumes were incorporated DMI and milk production increased. It is believed that a combination of improved nutritive value and increased DMI from grass-white clover (grass-clover) swards compared to grass-only swards results in increased milk production performance in dairy cows [9,17,18].

Many studies have reported increased lamb live weight gains from grass-clover swards compared to grazing older or predominantly grass-only swards [19,20]. Grace et al. [21] reported greater average daily weight gain, reduced days to target slaughter weight and increased carcass weight at slaughter for lambs grazing grass-clover swards compared to grass-only swards. Niderkorn et al. [22] examined the effect of increasing sward white clover content from 0 to 100% on the digestibility of grass-clover swards and DMI compared with grass-only swards. Those authors found no difference in DMI or sward digestibility when comparing mixed grass-white clover and grass-only, although DMI was significantly greater on pure white clover swards compared to pure grass swards.

Perennial ryegrass-only swards are known to vary in nutritive value due to seasonal and grazing management effects [23,24], subsequently affecting forage digestibility and DMI [25]. As the year progresses, fibre content of swards tends to increase and OMD, crude protein content and metabolizable energy content of swards tend to decline [23,25]. Grassland management can reduce some of the seasonal effects [24] by minimizing the proportion of stem in the sward. Swards with low forage digestibility, which tends to decline from spring to summer and be lower in autumn than spring, can reduce herbage DMI and subsequently reduce animal performance [24]. Grass-clover swards can maintain greater nutritive value for the total grazing season [26], increasing animal performance relative to grass-only in late summer and autumn. White clover growth peaks in late summer creating a complementary feed supply to the maturing grass and maintaining maximum productivity of the sward [27]. Although the seasonal effects on nutritive value are well known in grass-clover swards compared to grass-only swards [26,27,28], research assessing the total tract digestibility over a full grazing season of grass-clover forage is limited.

Sheep were used in the experiment reported in this paper as a model of the typical dairy cow [29] as they digest forage in a similar manner to cattle and are easier to manage due to their size compared to cattle [30]. The results can be used across sheep and dairy cows. The hypothesis of this study was that white clover inclusion in a grass sward would increase forage in vivo digestibility and voluntary DMI compared to grass-only forage in individually housed sheep in spring, summer and autumn. The objective of this study was to compare the digestibility and voluntary DMI of grass-only and grass-white clover (grass-clover) forage in individually housed sheep.

## 2. Materials and Methods

This experiment was approved by the Teagasc Animal Ethics Committee and authorized by the Health Products Regulatory Authority (HPRA) which is the competent authority in Ireland responsible for the implementation of European Union legislation (Directive 2010/63/EU) for the protection of animals used for scientific purposes.

This experiment was undertaken at Teagasc, Animal and Grassland Research and Innovation Centre, Moorepark, Fermoy, Co. Cork, Ireland (52°09′ N; 8°16′ W). Mean daily temperature during the experiment was 8.8, 15.1 and 12.9 °C and total rainfall was 35.8, 74.7 and 115.7 mm, respectively, for spring (April), summer (June/July) and autumn (September). The 10-year (2006–2017) average mean daily temperature was 8.8, 14.6 and 13.3 °C and rainfall was 58.8, 88.6 and 65.3 mm, respectively, for spring, summer and autumn. The soil type was free-draining acid brown earth of sandy loam-to-loam texture. In January 2017, two paddocks were selected on the research farm. The swards were predominantly perennial ryegrass-only and mixed perennial ryegrass and white clover. Both paddocks were reseeded in June 2013. The grass-only sward was sown with a 50:50 mix of perennial ryegrass cultivars Astonenergy (tetraploid) and Tyrella (diploid) at a seeding rate of 27.2 kg/ha, and the grass-clover sward had the same perennial ryegrass cultivars and sowing rate plus a 50:50 mix of Chieftain and Crusader medium leaf white clovers sown at 5 kg/ha.

The experiment was conducted with 12 one-year-old wether Texel sheep, six per treatment per period. The animals were weighed using an electronic portable weighing scales and the Winweigh software package (Trutest Limited, Auckland, New Zealand) prior to housing at each season and then randomly assigned to two groups which were balanced according to metabolic live weight (body weight (BW)^0.75^). Prior to, and in between, seasons the sheep were allowed graze predominantly grass-only swards with no supplementary feeding. Sheep were shorn in late May and received anthelmintic treatment in early June for internal parasites.

A 2 × 2 Latin square experimental design was used to investigate the effect of sward type on the in vivo digestibility in housed sheep. There were three experimental seasons and two measurement periods (P) in each season: spring (P1: 27 March–8 April, P2: 17–29 April), summer (P1: 19 June–1 July, P2: 10–22 July) and autumn (P1: 4–15 September, P2: 18–29 September). During P1 and P2 in the spring and summer seasons, animals were allowed to adapt to the diet and individual crates (adaptation phase) on days 1 to 6 and measurements were made on days 7 to 12 (measurement phase). In autumn the adaptation phase in P1 and P2 was 5 days and the measurement phase was from days 6 to 11 due to restricted availability of the shed facilitating the experiment.

The target herbage mass at harvest for each treatment in the spring, summer and autumn (>4 cm) was 1200–1600 kg DM/ha. Both paddocks were 0.38 ha in size and were sub-divided into four segments, randomly assigned per period, adaptation phase for P1 and P2 and measurement phase for P1 and P2 in each season. The target pre-cutting herbage mass for each period was manipulated using the sward regrowth interval. Regrowth estimation was calculated each week based on visual assessment. Between measurement seasons, the paddocks were mechanically harvested every 3–4 weeks.

Procedures described by Dermarquilly et al. [29] and Baumont et al. [31] were used to measure voluntary DMI and digestibility. Forage was offered in individual feed bins to the sheep for *ad libitium* consumption allowing a 10% refusal rate and adjusted daily as described by Beecher et al. [25]. Metabolic BW was used to estimate allowance on day 1 of the adaptation phase: forage offered (kg) = (BW^0.75^/1000) (kg) × 75 (g) × 110 (%)/forage DM (%) [32]. Forage was harvested daily at 08:30 with an Etesia mower (Etesia UK Ltd., Warick, UK). Average pre-cutting height was 10.8 cm and average post-cutting height was 4.17 cm and 4.24 cm on grass-only and grass-clover, respectively. Immediately after cutting approximately 50% of the daily forage allowance, based on the previous day’s allocation and refusal, morning forage was allocated to each sheep on a fresh matter basis. The evening allocation of forage was spread out on plastic in a cold room to avoid heating and stored at 4 °C. Once forage DM was calculated, evening allocation was offered on a DM basis to top up the morning allocation. The sheep were housed in individual stalls as described by Beecher et al. [25], allowing animals to be fed individually and allowing the total collection of faeces. Animals had free access to water and a salt lick at all times.

During P1 and P2 in each season, pre-cutting herbage mass was estimated twice weekly by harvesting one representative sample per treatment 4 cm above ground level (>4 cm) using a 0.25 cm^2^ quadrat and Gardena hand shears (Accu 60, Gardena International GmbH, Ulm, Germany). The sample was weighed and a subsample dried at 95 °C for 15 h in a forced convection oven (Parsons Lane, Hope Valley, UK) and used to calculate herbage mass (kg DM/ha). Immediately prior to harvesting, a 100 g forage sample was cut to ground level in the area about to be harvested for each treatment from days 6 to 11 in spring and summer and days 5–10 in autumn. The forage was divided into fractions above and below 4 cm from ground level. The proportion above 4 cm was separated into grass leaf, stem (including pseudostem) and dead, as described by Beecher et al. [7], and white clover for the grass-clover treatment, dried at 90 °C for 16 h in a forced convection oven (Parsons Lane, Hope Valley, UK) and weighed for DM proportions. Pre-cutting sward height and post-cutting sward height were determined daily in the area to be harvested that day in each treatment using a rising plate meter with a steel plate (Jenquip, Fielding, New Zealand) taking 10 measurements immediately before and after cutting.

Forage intakes and refusals were recorded daily during the measurement phase. Each morning refused forage from each sheep was weighed and sampled prior to cleaning the feed bins and allocating fresh forage to each sheep. For each treatment, the refused material was bulked and a 1 kg sub-sample removed for chemical analysis. Immediately after cutting, 1 kg sample of the fresh forage to be offered was collected from the harvested material for each treatment. Sub-samples for DM and chemical analysis were then collected from the refused and offered forage samples. Each day, 3 × 50 g of fresh offered and refused forage per treatment were dried at 120 °C for 4 h in a Gallenkamp Hotbox oven (Thermo Fisher Scientific INC., Waltham, MA, USA) for DM and used in the calculation of voluntary DMI. Approximately 200 g of fresh offered forage from each treatment for each day of the measurement phase was frozen at −18 °C and subsequently bowl-chopped (Muller, Type MKT 204 Special, Saabrücken, Germany), freeze-dried at −50 °C for 72 h and milled through a 1 mm screen. Forage samples from fresh offered and refused forage were dried at 60 °C for 48 h in a forced convection oven (Parsons Lane, Hope Valley, UK) and milled through a 1 mm screen using a Cyclotech 1093 Sample Mill (Foss, DK-3400 Hillerød, Denmark) and used later for chemical analysis.

Urine and faeces separation was facilitated by directing the urine through a funnel into a bucket directly below. Faecal collection trays were placed behind each sheep to allow for total daily faecal collection during the measurement phase. Trays were weighed, emptied and washed daily at 09:30. Once weighed, approximately 20% of the faeces was collected and frozen at −18 °C. From the defrosted faeces, approximately 15% of the original quantity of faeces produced per sheep per day per treatment was sub-sampled, weighed and oven-dried at 60 °C until completely dry and then milled, as described above, and stored for subsequent chemical composition analysis.

The quantity of fresh forage offered to and refused by each sheep was weighed daily to calculate DMI. Dry matter digestibility (DMD) was calculated once faeces were collected using the following equation:

DMD = (DMI (kg) − quantity of faeces excreted (kg DM))/(DMI (kg))(1)

The oven-dried samples of fresh offered forage, refused forage and faeces were tested for chemical composition using the same methods. The samples dried at 60 °C were analysed for ash by placing samples in a muffle furnace for 16 h at 550 °C ([33]; 942.05). The nitrogen (N) concentration was determined using the Dumas method (Leco FP-528; Leco Corporation, St., Joseph, MI, USA) adapted by Sweeney [34]. Crude protein (CP) was determined as N concentration × 6.25. Neutral detergent fibre (NDF) and acid detergent fibre (ADF; [33]; method 97318) were measured using the Ankom Fiber Analyser (Ankom Technology Corporation, Macedon, NY, USA) using the procedure of Van Soest et al. [35]. Sulphite was used in the NDF process to remove any protein remaining in the NDF residue [36], and ADF and NDF values are expressed including ash.

All data were analysed for normality using the univariate procedure of SAS 9.4 (SAS Institute Inc., Cary, NC, USA [37]). Sward data were analysed using the mixed procedure in SAS using the following model:

Y = µ+ Ti + (Pj)Sk + T × (P(S))ijk + e(2)

where µ = mean; Ti = treatment (I = 1, 2); Pj = period (j = 1, 2); Sk= season (k = 1, 2, 3); T × (P(S))ijk = the interaction of treatment and period within season; e = residual error term.

The DMI and digestibility data were analysed using the mixed procedure in SAS using the following model:

Y = µ+ Ti + (Pj)Sk + A(Ti)l + T × (P(S))ijk + e(3)

where µ = mean; Ti = treatment (I = 1, 2); Pj = period (j = 1, 2); Sk = season (k = 1, 2, 3); A(Ti)l = the effect of animal within treatment (l = 1…6); T × (P(S))ijk = the interaction of treatment and period within season; e = residual error term.

The model included season as the repeated measure. Animals were grouped by treatment and included as the random effect. The model specified the compound symmetry structure. For all data, the Tukey-Kramer multiple range test was used for mean separation (*p* < 0.05).

## 3. Results

### 3.1. Sward Characteristics

**Figure 1:**
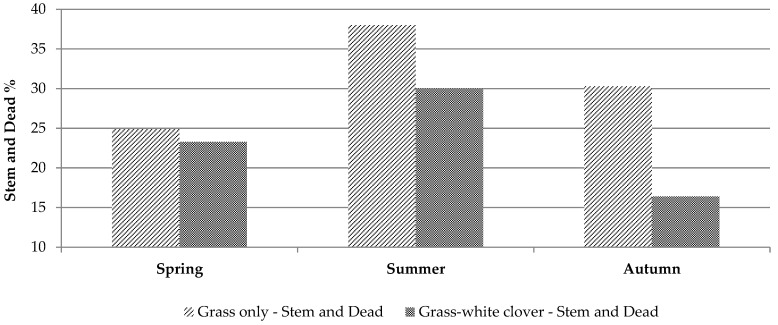
Combined stem and dead proportion of grass-only and grass-white clover swards offered to individually housed sheep in spring (27 March–29 April), summer (19 June–22 July) and autumn (4–29 September) (measured > 4 cm).

There was no effect of treatment or season on pre-cutting herbage mass (spring: 1378 and 1498 kg DM/ha on grass-only and grass-clover, respectively; summer: 1735 and 1790 kg DM/ha on grass-only and grass-clover, respectively; autumn: 1700 and 1553 kg DM/ha on grass-only and grass-clover, respectively, ±122 kg DM/ha). The regrowth period between harvests was 18–24 days. Measurements of sward morphology are presented in Table 1. Sward white clover content in the grass-clover swards was similar in spring (16.4%) and summer (17.0%), but significantly greater (*p* < 0.001) in autumn (35.6%). There was a significant treatment × season interaction on sward leaf (*p* < 0.001), stem (*p* < 0.01) and dead (*p* < 0.01) content. The grass leaf component was significantly greater (*p* < 0.001) in the grass-only sward than in the grass-clover sward in all seasons. The stem and dead components of the sward were similar for both treatments in spring and significantly different in summer and autumn (*p* < 0.01: Figure 1). The stem component of the sward was greatest in summer on both treatments and was least in autumn in the grass-clover sward. The dead component was similar in spring for both swards and lower on the grass-clover sward in summer and autumn compared to the grass-only sward.

### 3.2. Chemical Composition of the Offered Forage

**Figure 2:**
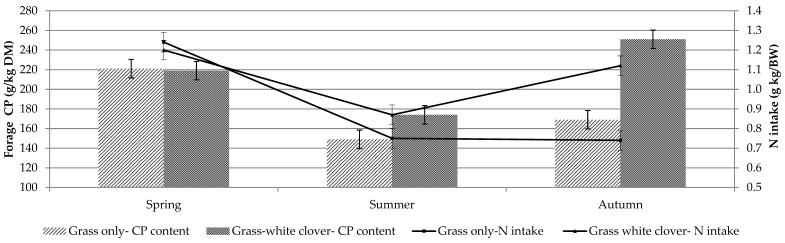
Forage crude protein content and daily N intake per kg body weight of grass-only and grass-white clover swards offered to individually housed sheep in spring (27 March–29 April), summer (19 June–22 July) and autumn (4–29 September) (measured > 4 cm).

**Figure 3:**
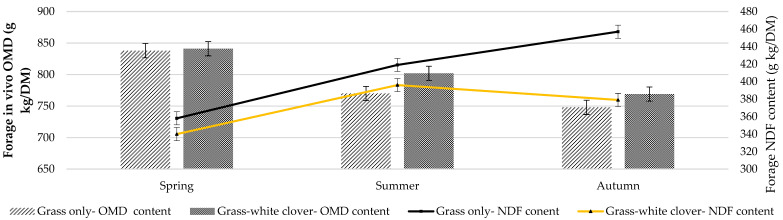
Forage in vivo OMD and forage neutral detergent fibre (NDF) concentration of grass-only and grass-white clover swards offered to individually housed sheep in spring (27 March –29 April), summer (19 June–22 July) and autumn (4–29 September) (measured > 4 cm).

**Figure 4:**
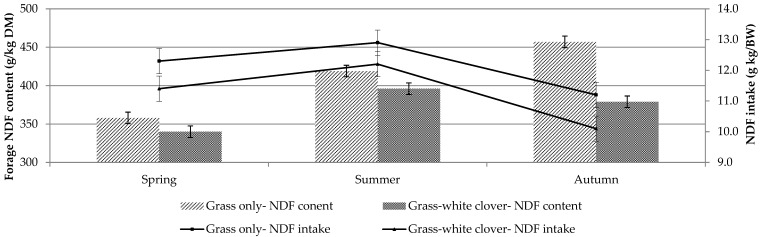
Forage NDF content and daily NDF intake of grass-only and grass-white clover swards offered to individually housed sheep in spring (27 March–29 April), summer (19 June–22 July) and autumn (4–29 September) (measured > 4 cm).

Crude protein and NDF concentration were similar between treatments in spring and summer (Table 2). In autumn, however, the grass-clover sward had significantly greater CP (Figure 2) and less NDF (Figure 3 and Figure 4) than the grass-only sward (*p* < 0.001; Table 2). Dry matter percentage was significantly affected by treatment and season. It was higher in grass-only than grass-clover (*p* < 0.001) and was highest in summer and least in autumn (*p* < 0.01). Season had a significant (*p* < 0.001) effect on both OM concentration, which was least in autumn and greatest in summer, and ADF concentration, which was least in spring and increased from spring to summer and summer to autumn.

### 3.3. Intake and Body Weight

Mean animal starting BW was 54.9 kg in spring, 65.5 kg in summer and 72.1 kg in autumn. Dry matter intake per day was greatest (*p* < 0.05) in summer and was similar between treatments (Table 3). Treatment had no effect on DMI per unit BW. Nitrogen intake (g/kg BW) was significantly affected by treatment, season and the treatment × season interaction (Table 3). Intake of N (g/kg BW) was similar for both treatments in spring and summer; however, in autumn, the grass-clover sward supported a significantly greater N intake than the grass-only (*p* < 0.001; Figure 3). Nitrogen intake was greatest in spring, decreased in summer and then stayed the same in autumn for grass-only. In contrast, N intake on the grass-clover treatment was greatest in spring, decreased in summer but then increased in autumn. Treatment and season had a significant effect on NDF intake (g/kg BW) (*p* < 0.001) (Table 3; Figure 4), which was consistently less on the grass-clover sward compared to grass-only in all seasons and greatest in summer and least in autumn for both treatments. Season had a tendency (*p* < 0.07) to affect ADF intake, which was greatest in summer for both treatments. Acid detergent fibre intakes were similar across all treatments. Treatment had no effect on digestible organic matter intake (DOMI) or OMI but they were affected by season. Both were greatest in spring and least in autumn (*p* < 0.001).

### 3.4. Digestibility of the Swards

Organic matter digestibility (Figure 3) and DMD were greater for grass-clover forage compared to grass-only (*p* < 0.05) (Table 4). Dry matter digestibility and OMD were greatest in spring and least in autumn (Table 4). Treatment had a significant effect on N digestibility, which was always greater on the grass-clover sward (*p* < 0.001) compared to grass-only. The N digestibility was greatest in spring and least in summer (*p* < 0.001). Season had a significant effect on NDF and ADF digestibility; both were greatest in spring (Table 4). Generally, as forage NDF content increased, forage in vivo digestibility declined (Figure 3).

## 4. Discussion

The hypothesis of this experiment was that the inclusion of white clover into a grass sward would increase the forage in vivo digestibility and voluntary DMI of individually housed sheep in spring, summer and autumn. White clover inclusion increased forage OM and DM digestibility across all seasons in comparison to the grass-only forage, and thus part of the hypothesis is accepted. However, the digestibility increases on the grass-clover forage were generally small (0% for NDF, 2–3% for DM, OM, ADF, and 12% for N), and, therefore, may explain why white clover inclusion had no significant effect on voluntary DMI in sheep in comparison to the grass-only forage; therefore, there is insufficient data from this study to support this part of the hypothesis.

Overall, including clover in the sward reduced the leaf, stem and dead components of the perennial ryegrass part of the grass-clover sward. However, the leaf, stem and dead proportions of the grass component of the grass-clover sward were similar to the grass-only sward. Therefore, it is likely that the grass component of the grass-clover sward had digestibility similar to that of the grass-only sward, which was similar to that reported by Beecher et al. [25]. Importantly, this shows that even with good quality grass, incorporating clover has a positive effect on the forage quality offered to livestock through the improved digestibility of that forage. This is important to pasture-based systems as it increases the potential nutrient intake, which can lead to increased animal production [9,23,25]. Clover has lower NDF and ADF and greater CP concentration than the ‘whole’ perennial ryegrass plant [22]. While the inclusion of clover reduced the proportion of grass leaf in the sward in all seasons, the most digestible component of the grass-clover sward (total leaf plus clover) increased by 12.7% in summer and 20% in autumn compared to the grass-only sward, and conversely reduced stem and dead content. Increasing the nutritionally superior components in the sward was reflected in the higher CP concentration and lower NDF concentrations in the grass-clover sward compared to the grass-only sward in autumn. One of the reasons for the higher CP content in both forages in spring compared to summer and compared to grass-only in autumn may be linked to pre-cutting herbage mass. As herbage mass increases, sward CP concentration tends to decline due to a dilution effect [38].

Sward clover proportion was similar in the spring and summer in this study but was less than the average in summer reported by McClearn et al. [18] (250 g/kg DM) and Egan et al. [14] (200 g/kg DM). Sward clover proportion was greatest in autumn similar to Schils et al. [39] and Egan et al. [14,27,40]. Including clover into the sward was nutritionally beneficial as there was a 49% increase in CP and 17% reduction in NDF concentration in the grass-clover sward compared to the grass-only sward in autumn. The increased forage quality of the grass-clover sward in autumn resulted in forage that was similar in chemical composition to that of the spring grass-only. This is an important consequence of including clover in grass swards as the quality of grass can decline in autumn, negatively impacting animal performance [26,28,41].

It is well established that dairy cow DMI is greater on grass-clover swards than grass-only swards [9,26,27], resulting in increased animal performance [41,42]. The greater palatability and lower resistance to particle breakdown of clover compared to grass [43,44] can potentially encourage greater intakes of grass-clover swards compared to grass-only [45] seen in dairy cows [18,26,27] but not observed in this experiment. There was no effect of clover inclusion into grass swards on daily DMI or DMI per kg BW in this study. Although not significantly different, clover inclusion increased sheep daily DMI by 13% compared to the grass-only treatment in autumn. Penning et al. [46] found significant DMI differences in lactating ewes compared to non-lactating ewes and concluded that the nutritional demand differences in ewes dictated DMI differences. The sheep used in this study increased in BW from spring (54.9 kg) to summer (65.5 kg) to autumn (72.1 kg) and were heavier than sheep used by Beecher et al. [25] (58 kg) and [22] (52 kg) and that recommended by Dermarquilly et al. [29] (60 kg) for in vivo digestibility studies. In this study, DMI was greater than that reported by References [22,25] as sheep weight was greater in this study. As the sheep increased in BW from spring to summer to autumn, they grew closer to their mature BW and, therefore, their energy requirements for growth declined [47]. It is likely that in the autumn measurement period their energy intake was only to satisfy maintenance requirements and hence the reduced OMI and DOMI.

The greater CP concentration in autumn and greater N digestibility in summer and autumn of the grass-clover sward compared to the grass-only sward significantly increased N intake per sheep per unit BW in the current study. Intensive pasture-based swards often contain greater concentrations of N than required by ruminants, which can have negative implications for environmental sustainability and animal production [48,49] because the excess N not utilised is excreted. The nutritive value of grass is highest in spring [23], hence the highest OM and N intakes for both treatments were observed in spring of this study. The CP concentration in the grass-clover sward was highest in autumn, resulting in increased N intake on grass-clover compared to grass-only. The grass-clover had lower NDF concentration compared to grass-only, resulting in reduced NDF intake per unit BW in spring (7%), summer (6%) and autumn (10%) compared to grass-only. The increased rainfall in autumn resulted in low herbage DM concentration in both swards, possibly reducing the difference in intake, especially when herbage quality was high in both swards.

The incorporation of clover into a grass sward can increase the digestion of the soluble fraction or the most digestible components of the diet (e.g., [50]). A full system dairy cow grazing experiment by Guy et al. [28] found increases in forage OMD when clover was included in the grass sward, similar to this study. Adding clover maintained greater in vivo OMD and DMD concentrations in summer and autumn compared to the grass-only sward in this study. A previous in vivo digestibility study reported that OMD explained 36% of the variation in voluntary DMI [25]. This study showed a 4% and 3% increase in OMD, increasing sheep DMI by 10 and 280 g per day in summer and autumn, respectively, on the grass-clover sward compared to the grass-only sward. The lower increase in DMI in summer compared to Beecher et al. [25] may have been due to the low sward clover content in summer, resulting in fewer differences in chemical composition between swards and compared to autumn and the size of sheep as mentioned previously.

## 5. Conclusions

Clover inclusion into the grass sward reduced grass stem and dead proportions compared to the grass-only sward. An increased proportion of digestible and higher nutritive value components in the grass-clover sward (leaf and clover) increased in vivo OMD and DMD in summer and autumn and increased CP concentrations in autumn compared to the grass-only sward. Lower NDF concentration in the grass-clover sward reduced total NDF intake per sheep per unit BW in all seasons compared to the grass-only swards. Clover did not affect spring forage chemical composition, DMI or digestibility. However, in autumn, the grass-clover sward had higher CP concentration and similar NDF concentration to that seen in grass-only spring forage. This, combined with the increased OMD in autumn, could potentially improve animal production compared to a grass-only sward.

## Figures and Tables

**Table 1:** Morphology of the forage offered to individually housed sheep in spring (27 March–29 April), summer (19 June–22 July) and autumn (4–29 September) (measured >4 cm). LSMeans for the interaction are presented.

	Spring		Summer		Autumn			Level of Significance		
	Grass-Only ^†^	Grass-Clover ^‡^	Grass-Only	Grass-Clover	Grass-Only	Grass-Clover	S.E.M. ^§^	Treatment	Season	Treatment × Season
Leaf proportion (g/kg DM)	751 ^a^	603 ^b^	621 ^b^	530 ^c^	697 ^d^	481 ^e^	14.5	<0.001	<0.001	<0.001
Stem proportion (g/kg DM)	172 ^bc^	155 ^c^	259 ^a^	202 ^b^	164 ^bc^	74 ^d^	14.1	<0.001	<0.001	<0.01
Dead proportion (g/kg DM)	77 ^c^	78 ^bc^	120 ^a^	98 ^b^	139 ^a^	90 ^bc^	7.2	<0.001	<0.001	<0.01
White clover proportion (g/kg DM)	-	164 ^b^	-	170 ^b^	-	356 ^a^	10.5	-	<0.001	-

^a–e^ Means within a row without a common superscript differ (*p* < 0.05). ^†^ Perennial ryegrass–only sward receiving 250 kg N/ha/year; ^‡^ Perennial ryegrass-white clover sward receiving 150 kg N/ha/year; ^§^ S.E.M.—standard error mean.

**Table 2:** Sward chemical composition of the forage offered to individually housed sheep in spring (27 March–29 April), summer (19 June–22 July) and autumn (4–29 September) (measured >4 cm). LSMeans for the interaction are presented.

	Spring		Summer		Autumn			Level of Significance		
	Grass-Only ^†^	Grass-Clover ^‡^	Grass-Only	Grass-Clover	Grass-Only	Grass-Clover	S.E.M. ^§^	Treatment	Season	Treatment × Season
DM (g/kg)	176 ^bc^	167 ^c^	206 ^a^	187 ^b^	131 ^d^	111 ^e^	5.5	<0.01	<0.001	<0.05
OM (g/kg DM)	912 ^b^	911 ^b^	925 ^a^	926 ^a^	909 ^bc^	900 ^c^	3.3	n.s. ^¶^	<0.001	<0.05
CP (g/kg DM)	221 ^b^	219 ^b^	149 ^c^	174 ^c^	169 ^c^	251 ^a^	9.4	<0.001	<0.001	<0.001
NDF (g/kg DM)	358 ^c^	340 ^d^	419 ^b^	396 ^c^	457 ^a^	379 ^c^	7.5	<0.001	<0.001	<0.001
ADF (g/kg DM)	199	195	233	233	269	259	5.3	n.s.	<0.001	n.s.

^a–d^ Means within a row without a common superscript differ (*p* < 0.05). ^†^ Perennial ryegrass–only sward receiving 250 kg N/ha/year; ^‡^ Perennial ryegrass-white clover sward receiving 150 kg N/ha/year; ^§^ S.E.M.—standard error mean; ^¶^ n.s.—not significant.

**Table 3:** Effect of forage type (grass-only and grass-clover) offered to individually housed sheep in spring (27 March–29 April), summer (19 June–22 July) and autumn (4–29 September) on voluntary dry matter intake (DMI) per body weight (BW), digestible organic matter intake (DOMI) and intake of chemical components. LSMeans for the interaction are presented.

	Spring		Summer		Autumn			Level of Significance		
	Grass-Only ^†^	Grass-Clover ^‡^	Grass-Only	Grass-Clover	Grass-Only	Grass-Clover	S.E.M. ^§^	Treatment	Season	Treatment × Season
DMI (kg/d)	1.98	1.98	2.04	2.05	1.78	2.02	0.081	n.s. ^¶^	<0.05	n.s.
DMI (g/kg BW)	35.3	34.7	31.2	31.3	28.0	24.8	1.03	n.s.	<0.001	0.1286
OMI (g/kg BW)	32.3	31.7	29.3	29.5	22.3	25.3	0.93	n.s.	<0.001	n.s.
DOMI (g/kg BW)	27.4	26.7	23.6	22.6	16.7	19.5	0.84	n.s.	<0.001	n.s.
NI (g/kg BW)	1.24 ^a^	1.20 ^a^	0.75 ^b^	0.87 ^b^	0.74 ^b^	1.12 ^a^	0.05	<0.001	<0.001	<0.001
NDFI (g/kg BW)	12.3	11.4	12.9	12.2	11.2	10.1	0.41	<0.01	<0.001	n.s.
ADFI (g/kg BW)	6.9	6.7	7.2	7.3	6.5	7.1	0.25	n.s.	n.s.	n.s.

^a,b^ Means within a row without a common superscript differ (*p* < 0.05). ^†^ Perennial ryegrass–only sward receiving 250 kg N/ha/year; ^‡^ Perennial ryegrass-white clover sward receiving 150 kg N/ha/year; ^§^ S.E.M.—standard error mean; ^¶^ n.s.—not significant.

**Table 4:** Effect of season (spring (27 March–29 April), summer (19 June–22 July) and autumn (4–29 September)) and forage type offered (grass-only or grass-clover) to individually housed sheep on the digestibility of nutrient components.

Digestibility (g/kg)	Spring	Summer	Autumn	S.E.M.	*p*-Value	Grass-Only ^†^	Grass-Clover ^‡^	S.E.M. ^§^	*p*-Value
DM	814 ^a^	758 ^b^	728 ^c^	6.31	<0.05	756 ^b^	778 ^a^	6.31	<0.001
OM	840 ^a^	786 ^b^	759 ^c^	8.01	<0.05	785 ^b^	804 ^a^	6.54	<0.001
N	783 ^a^	685 ^b^	723 ^b^	13.35	<0.001	670 ^b^	761 ^a^	10.90	<0.001
NDF	787 ^a^	706 ^b^	698 ^b^	11.82	<0.001	731	730	9.65	n.s. ^¶^
ADF	692 ^a^	645 ^b^	654 ^ab^	12.96	<0.05	656	671	10.59	n.s.

^a–c^ Means within a row without a common superscript differ (*p* < 0.05). ^†^ Perennial ryegrass–only sward receiving 250 kg N/ha/year; ^‡^ Perennial ryegrass-white clover sward receiving 150 kg N/ha/year; ^§^ S.E.M.—standard error mean; ^¶^ n.s.—not significant.

## Data Availability

Not applicable.
